# Confirmation of Organized Modularity in the Yeast Interactome

**DOI:** 10.1371/journal.pbio.0050153

**Published:** 2007-06-12

**Authors:** Nicolas Bertin, Nicolas Simonis, Denis Dupuy, Michael E Cusick, Jing-Dong J Han, Hunter B Fraser, Frederick P Roth, Marc Vidal

A recent *PLoS Biology* article [1] rejected the conclusions of two previous publications [2,3] that two categories of highly connected “hub” proteins—“date” and “party” hubs—have distinct properties in the Saccharomyces cerevisiae interactome network. Currently available protein–protein interaction datasets are vastly incomplete, even for yeast [4]. Therefore, it is reasonable to rigorously re-scrutinize global properties of interactome networks as new datasets become available. Here we show that distinctions between date and party hubs [2], previously shown in a high-quality filtered yeast interactome (FYI) dataset [2,3], are in fact confirmed in an updated literature-curated yeast interactome network.

## Data Quality

Two protein–protein interaction datasets were used in [1]: a high-confidence (HC) network obtained from both curated literature and high-throughput sources, and a subgraph of HC that was obtained by linking the nodes of FYI with HC edges (HC^fyi^). As explained in [2], it is crucial that high-quality data be used to partition date and party hubs. Therefore, FYI was originally generated as the union of two high-confidence interaction datasets: one curated from small-scale studies published in the literature [5] and another obtained by stringently requiring support from at least two out of four sources of high-throughput interaction evidence [2]. We use a similar definition here to derive a filtered high-confidence (‘filtered-HC’) dataset containing 2,561 proteins linked by 5,996 interactions (Table S1) from HC. To eliminate false positive interactions that were either reported once but never confirmed or that were obtained through curation error, our analysis included literature-curated interactions only if they were observed in two independent articles (i.e., associated with two or more independent PubMed IDs). Moreover, many interactions in HC were derived from a single experiment reported in multiple publications—e.g., reference [6] describes an approximate superset of the experiments including those reported in reference [7]. Such publications [6–10] were considered dependent and merged. Thus 2,423 protein pairs were removed from HC. Also, we did not include interactions supported solely by high-throughput yeast two-hybrid screening [11,12] (97 pairs) or supported solely by high-throughput pull-down followed by mass spectrometry screening (742 pairs) [6–10,13] (see Table S1 for a complete list of interactions in filtered-HC).

## Consistency of Date and Party Hub Classification across Datasets

We identified date and party hubs in both HC and filtered-HC (all analyses were also performed on the HC^fyi^ network; see Figure S1). Since both networks contain many new interactions relative to FYI, and since some erroneous interactions might have been corrected, the proteins originally identified as hubs in FYI cannot and should not be assumed to be identical. For the analyses described here, we therefore defined hubs anew using a degree threshold that includes the top 20% most connected nodes [2]. This corresponds to a degree of 10 or more for HC (19.4% of the proteins) and a degree of 7 or more for filtered-HC (21.7%).

In the original report of the date/party hub distinction [2], bimodality was observed in the average Pearson's correlation coefficient (AvgPCC) distribution of hubs for two out of five expression datasets examined [2]. The complete lack of bimodality observed in [1] may stem from a conservative statistical test that assumed a uniform unimodal null distribution. We emphasize that bimodality was not deemed essential evidence of the party/date hub distinction in the initial report [2].

**Figure 1:**
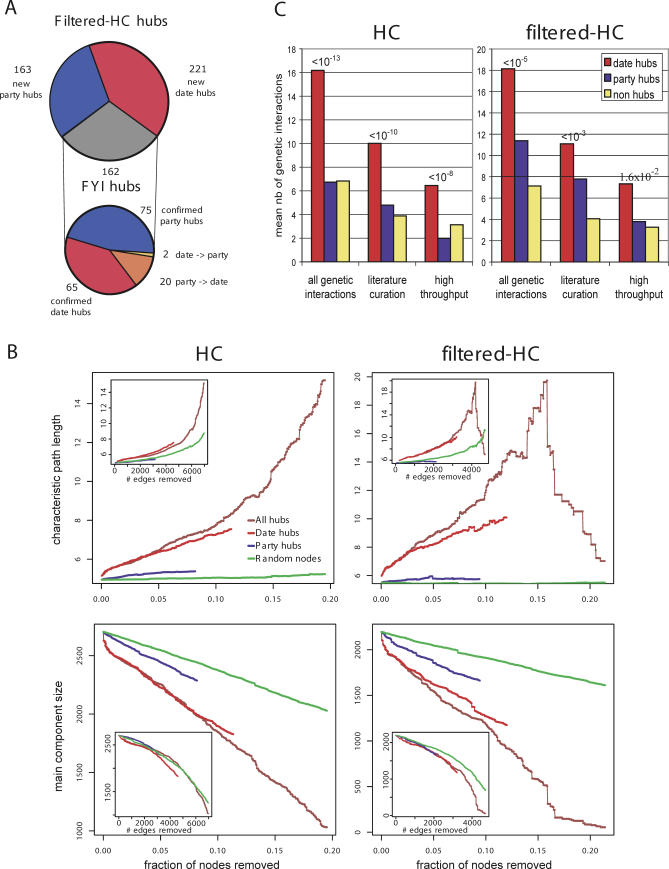
Analysis of Hub Properties in the Filtered-HC and HC Interactome Networks (A) Consistency of the party/date attribution between FYI and filtered-HC. Because filtered-HC network has many more interactions than FYI, only 162 of the 546 hubs in filtered-HC had been previously found in FYI. Filtered-HC confirmed 86% of the party/date designations in FYI. In addition, 20% of FYI hubs are not considered as hubs anymore in the new filtered-HC network because of the higher connectivity threshold. (B) The effect on the characteristic path length (top panels) and main component size (bottom panels) of the networks upon gradual node removal for HC (left panels) and filtered-HC (right panels). Attacks against all hubs (brown curve), party hubs (blue curve), date hubs (red curve), and random nodes (green curve). Insets show an additional control for connectivity differences between categories with the x-axis representing the number of edges removed from the network. (C) Date hubs participate in more genetic interactions than party hubs or non-hubs [2], as measured here by mean number of interactions [1] from a network of curated genetic interactions [32] for both filtered-HC (right panel) and HC (left panel). Inside each panel, bars show the number of genetic interactions held by date hubs (red), party hubs (blue), and non-hub proteins (yellow). The *p*-values assessing the difference of the means between date and party hubs (Mann-Whitney *U*-test) are indicated above the bars.

Since party and date hubs fall along a continuum, the choice of an AvgPCC threshold that distinguishes them is somewhat arbitrary (although our previous conclusions were robust to this choice [2]). Therefore, we adopted the PCC threshold of 0.5 for all networks considered here (this is the same threshold applied previously to PCC distributions that did not appear bimodal [1,2]). Thirteen expression datasets [14–31] were considered in addition to the original five independent datasets [2] (see Table S2). Strikingly, 86% of the FYI-defined hubs found in filtered-HC retained their date/party designation (Figure 1A) (81% for HC). This indicates that assignment to one or the other category is robust across datasets.

We suggest that some analyses presented in [1] (in particular the network tolerance to hub deletion) erred by not taking into account new hubs defined by the increased number of interactions relative to the original FYI. This strategy ignores 46% of the hubs in HC^fyi^ [1] and thus effectively immunizes them in the attack resistance analysis and eliminates them from the genetic interaction comparison.

## Distinct Topological Properties of Date and Party Hubs

When removed from the network, party and date hubs have strikingly distinct effects on the overall topology of HC, filtered-HC, and HC^fyi^. Removing date hubs dramatically disrupts the characteristic path length (CPL) of the network, whereas removing party hubs has a negligible effect (Figure 1B), as previously observed [2]. Importantly, this difference in behavior is not sensitive to the specific threshold values of degree *k* and AvgPCC chosen here to define hubs and party hubs, respectively (Figure S2). The CPL of a network measures the mutual closeness of nodes in a network. The claim in [1] that date and party hub removal has an indistinguishable effect on network topology was based on the analysis of a different topological feature altogether—main component size. This is a poor measure of network clustering in that it does not, for example, discriminate an extended beads on a string topology from a completely connected clique. This measure is also highly sensitive to a single spurious interaction that connects two otherwise disconnected subgraphs. By contrast, the dramatic decrease in CPL that we observe for date hubs in HC, filtered-HC, and HC^fyi^ suggests their coordinating role and confirms the original findings [2].

## Genetic Interactions

In [2] we showed that date hubs exhibit a higher genetic interaction density than party hubs. Reference [1] described analysis of two sets of genetic interactions: one from a union of high-throughput studies (HTP-GI), and another from the literature (LC-GI) [32]. Both LC-GI and HTP-GI datasets are potentially subject to bias since gene pairs were selected nonrandomly for testing, but these are the best datasets currently available. While the LC-GI analysis confirmed our original finding, the HTP-GI analysis did not [1], which we confirmed using date/party hubs defined from FYI. However, examining HTP-GI in the larger HC and filtered-HC networks, we find that date hubs in both HC and filtered-HC exhibit higher genetic interaction density than party hubs or non-hubs (Figure 1C), confirming the original report [2]. This difference remains after controlling for connectivity of hubs in the protein interaction network (Figure S3).

## Evolutionary Rate

We also confirmed the difference in evolutionary rates [33] between date and party hubs that was reported previously [3]. Using the filtered-HC network (with hubs defined as above) we found that date hubs evolve significantly faster than party hubs (Wilcoxon *p* = 0.01). Furthermore, using our expanded expression dataset, the PCC of hubs was negatively correlated with their evolutionary rates (Pearson *r* = −0.22, *p* = 1 ×10^−7^), even when controlling for protein abundance [34] in either rich (Pearson partial *r* = −0.19, *p* = 3 ×10^−6^) or minimal media (Pearson partial *r* = −0.20, *p* = 2 ×10^−6^). The same result was obtained when considering the HC and HC^fyi^ networks (unpublished data). Moreover, a recent report independently supported evolutionary rate differences between date and party hub and explained these differences in terms of three-dimensional protein structure [35].

## Summary

We confirmed that date and party hubs have different topological properties, with the coordinating role of date hubs being supported by a greater impact on CPL. We also confirmed that date hubs participate in more genetic interactions and evolve more rapidly than party hubs. These observations, as well as the identity of the nodes considered as date and party, remained largely consistent within all tested networks (HC, filtered-HC, HC^fyi^), demonstrating the robustness of the results originally observed in [2]. Thus, this updated analysis confirms the validity of the distinction between date and party hubs in the yeast interactome [2,3], and shows that the date and party hub concept and the “stratus-like” network [1] model are not mutually exclusive.

## Supporting Information

Figure S1 Hub Deletion and Genetic Interaction Analysis for the HC^fyi^ Interaction Network as Defined in [1] (172 KB PDF).

Figure S2 Different Effect on Gradual Date or Party Node Removal on the CPLs of the Networks for Filtered-HC Is Not Dependent on the PCC Threshold Used to Define Party Hubs. (378 KB PDF).

Figure S3 Genetic Connectivity of Date and Party Hubs (A) Mean number of genetic interactions reported corrected by the physical connectivity. (B) The mean absolute connectivity for each hub category and the genetic interaction connectivity normalized by the number of protein–protein interactions observed for all three protein–protein interaction datasets using either HTP-GI or LC-GI separately or combined. *p*-values assessing the difference of the means (Mann-Whitney *U*-test) are indicated.
(102 KB PDF).

Table S1 Filtered-HC Protein-Protein Interaction Dataset. (8.5 MB XLS).

Table S2 Filtered-HC Date and Party Hubs Degrees, Clustering Coefficients and AvgPCC Values for Each Microarray Dataset. (252 KB XLS).
