# Supplementary material for: Confirmation of Organized Modularity in the Yeast Interactome
Source: PLoS Biol. 2007 Jun 12;5(6):e153. doi: 10.1371/journal.pbio.0050153 (PMC1892830; doi:10.1371/journal.pbio.0050153)

HC^{fyi} topological features and genetic interactions density

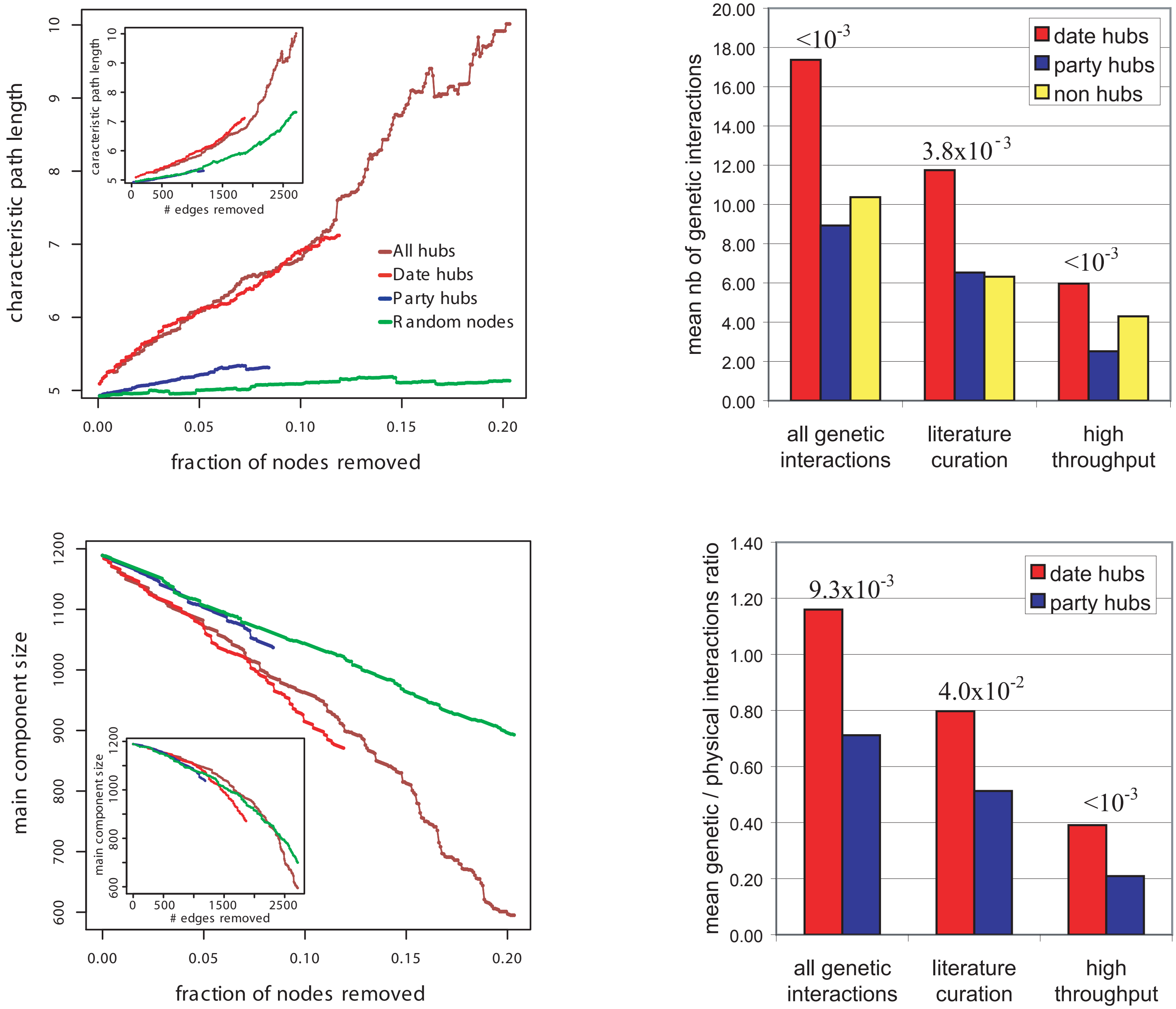

Supplement: Supplementary file 1 [file pbio.0050153.sg001.pdf]
