# Supplementary material for: Confirmation of Organized Modularity in the Yeast Interactome
Source: PLoS Biol. 2007 Jun 12;5(6):e153. doi: 10.1371/journal.pbio.0050153 (PMC1892830; doi:10.1371/journal.pbio.0050153)

Filtered-HC PCC thresholds sampling

PCC threshold 0.4

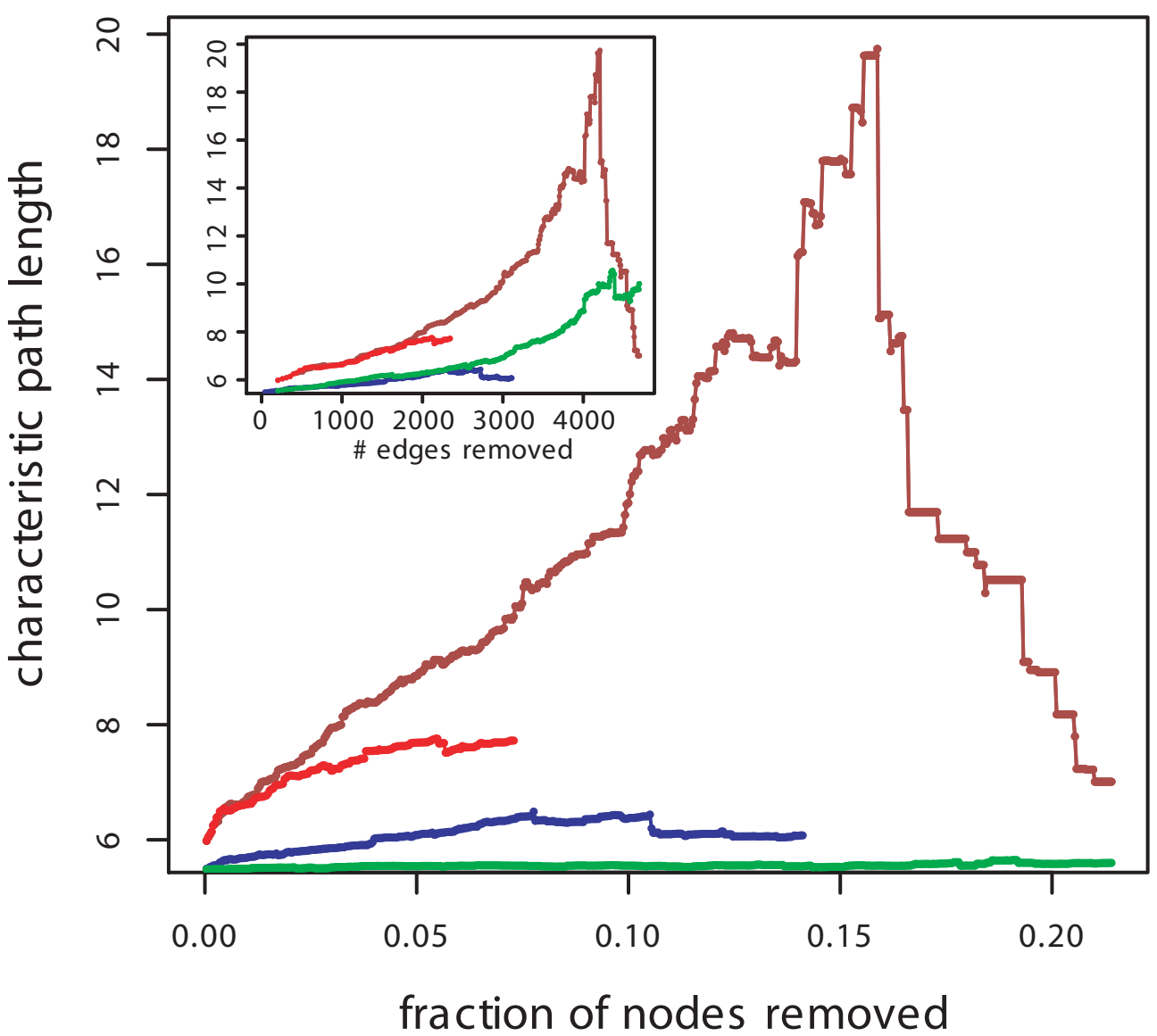

PCC threshold 0.45

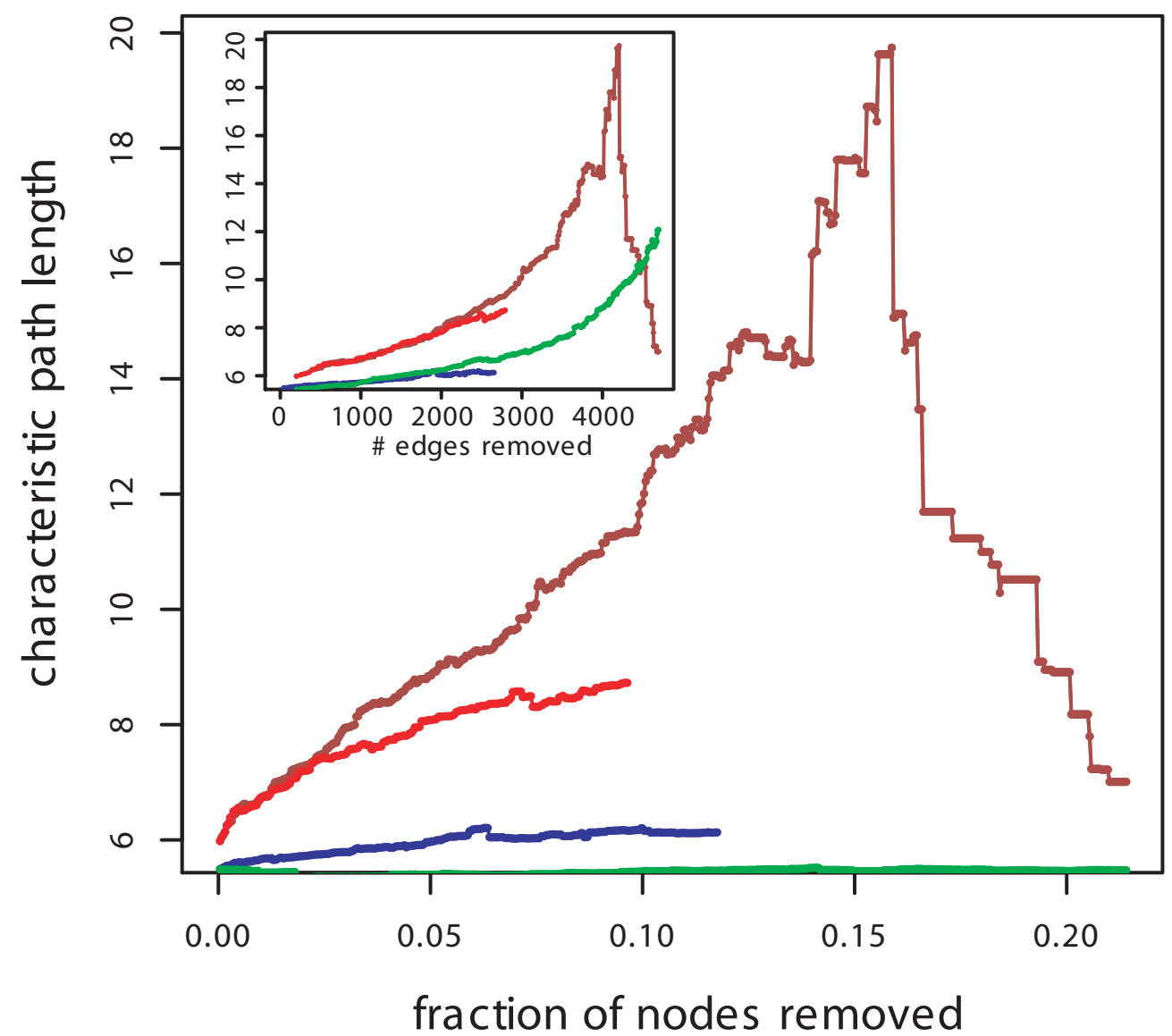

PCC threshold 0.5

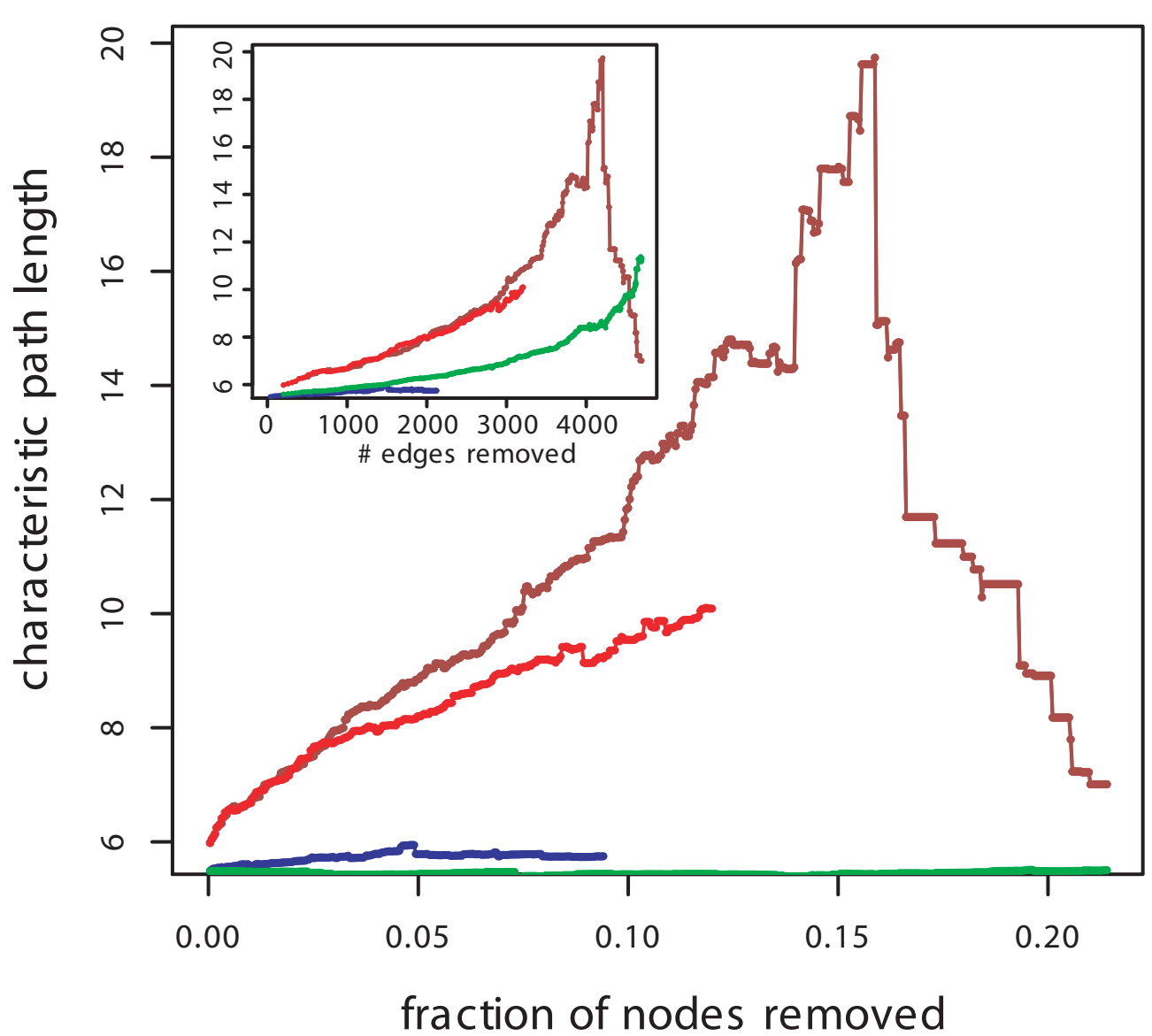

PCC threshold 0.55

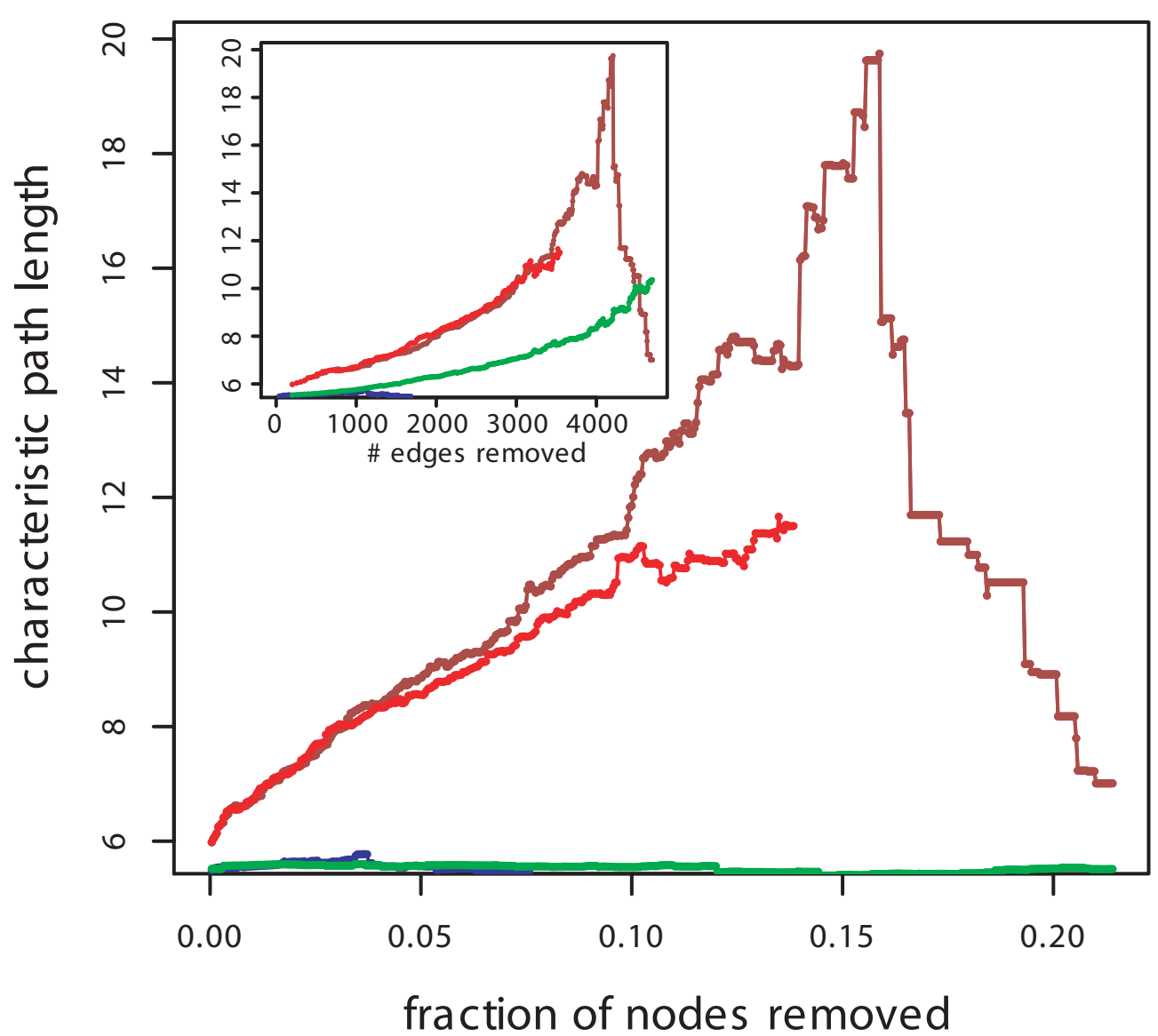

PCC threshold 0.6

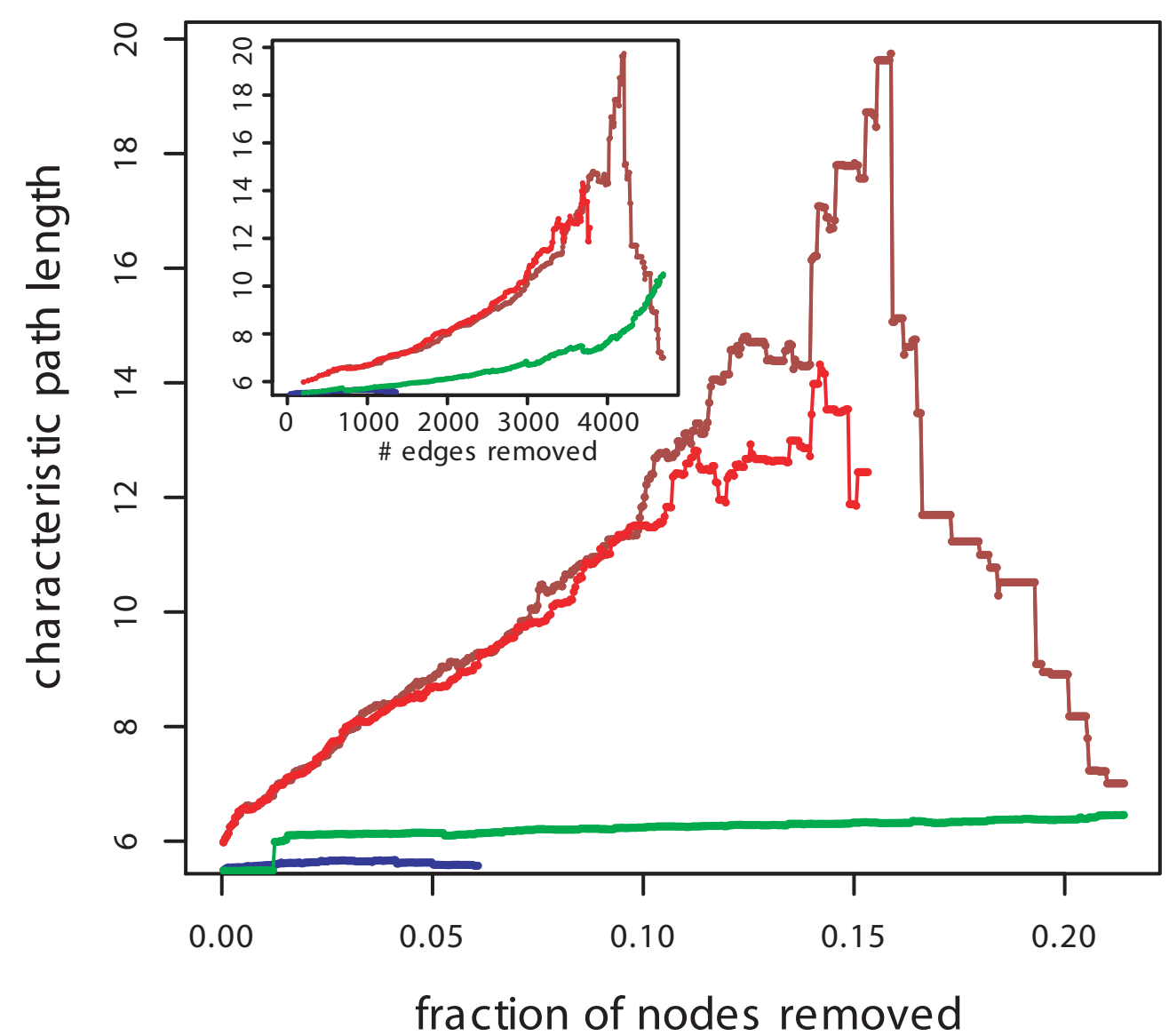

Supplement: Supplementary file 2 [file pbio.0050153.sg002.pdf]
