# Supplementary material for: Confirmation of Organized Modularity in the Yeast Interactome
Source: PLoS Biol. 2007 Jun 12;5(6):e153. doi: 10.1371/journal.pbio.0050153 (PMC1892830; doi:10.1371/journal.pbio.0050153)
Supplement: Supplementary file 3 [file pbio.0050153.sg003.pdf]

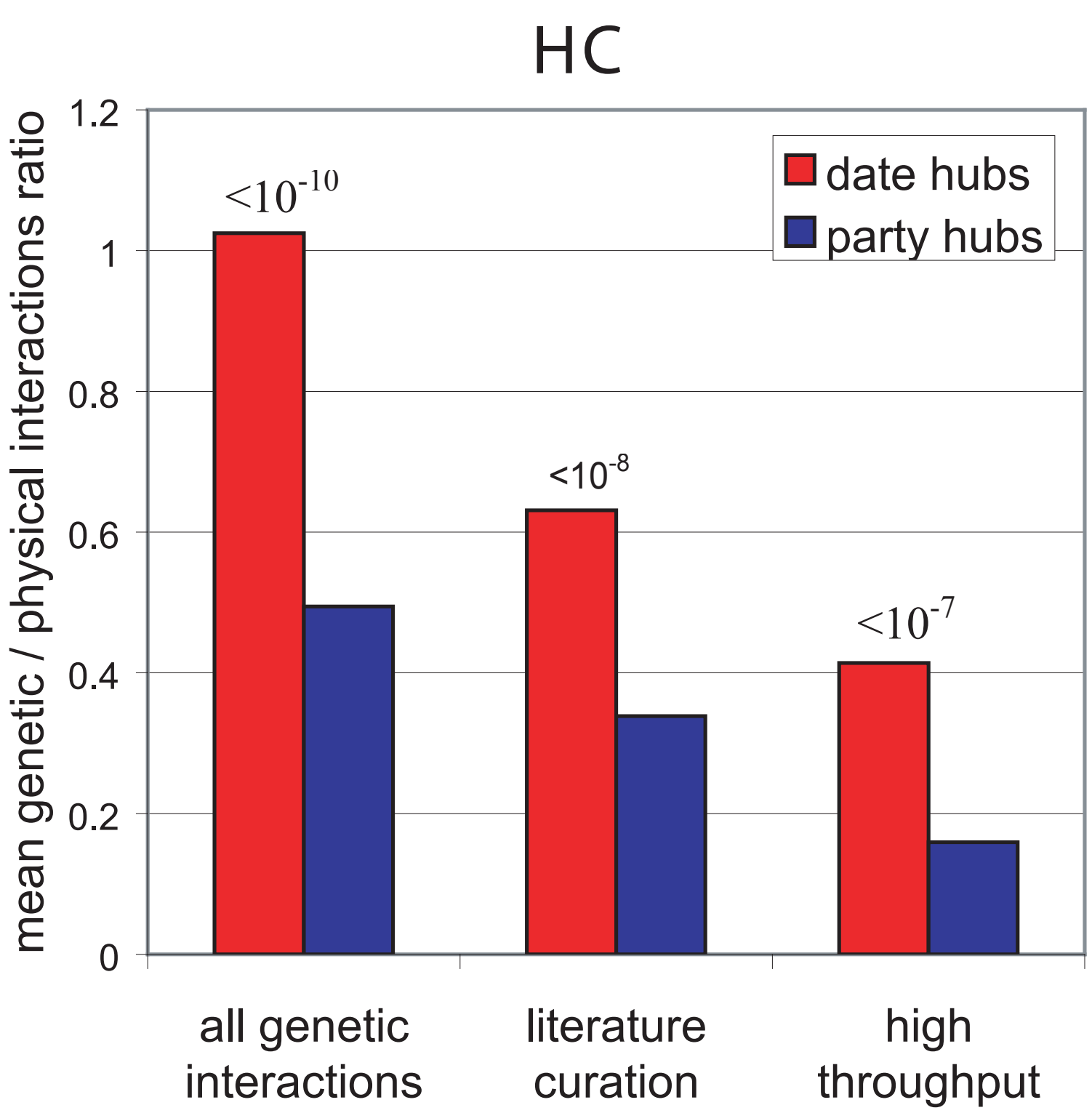

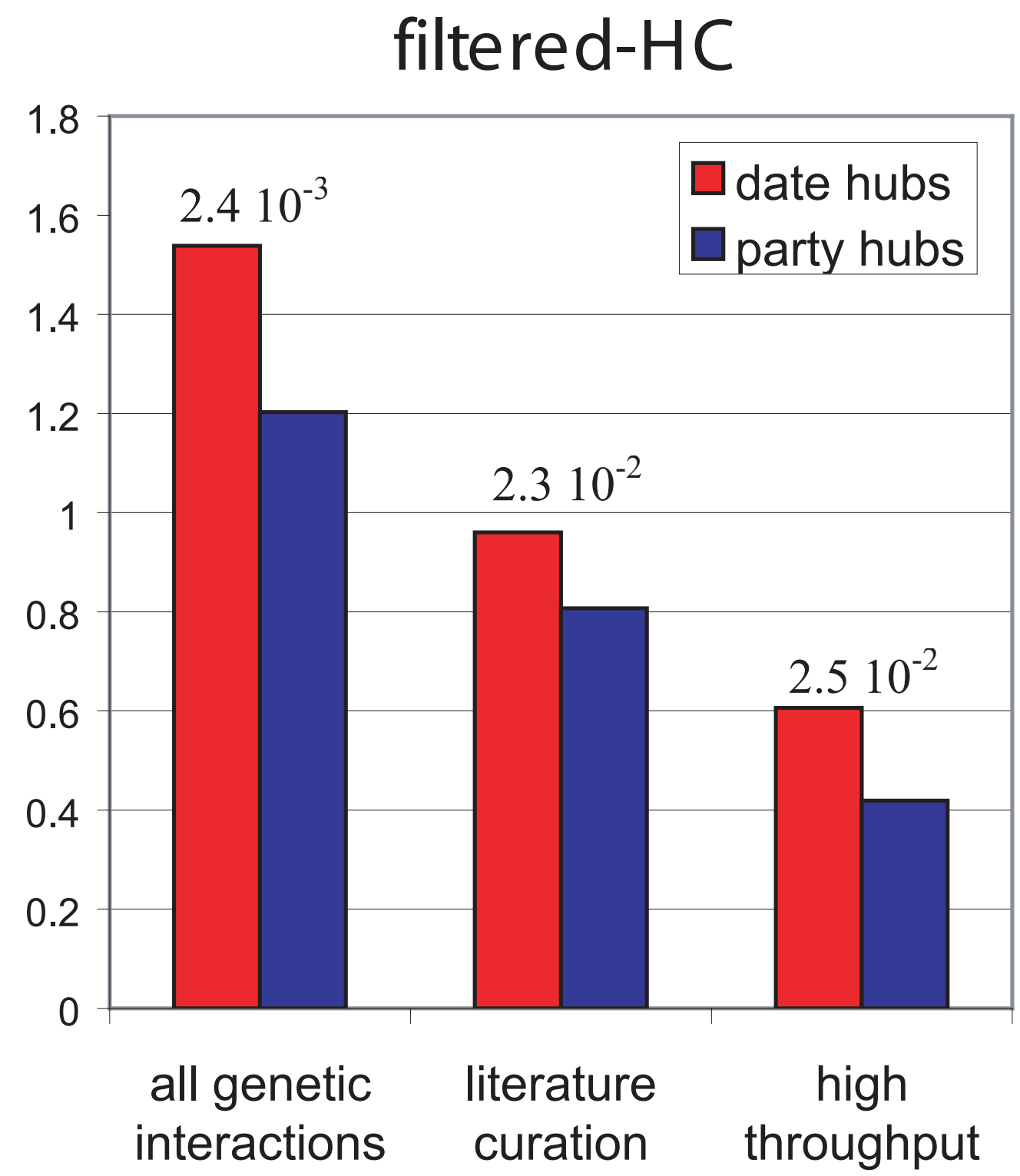

**HC**	date	party	p-value	date corrected	party corrected	p-value corrected
all genetic interactions	16.18	6.74	8.6E-14	1.02	0.49	1.1E-11
literature curation	10.02	4.80	5.1E-11	0.63	0.34	3.3E-09
high throughput	6.45	1.99	8.4E-09	0.41	0.16	1.5E-08
**HC filtered**	date	party	p-value	date corrected	party corrected	p-value corrected
all genetic interactions	18.14	11.39	6.4E-06	1.54	1.20	2.4E-03
literature curation	11.10	7.79	1.7E-04	0.96	0.81	2.3E-02
high throughput	7.34	3.80	1.6E-02	0.61	0.42	2.5E-02
**HC fyi**	date	party	p-value	date corrected	party corrected	p-value corrected
all genetic interactions	17.37	8.93	9.7E-04	1.16	0.71	9.3E-03
literature curation	11.75	6.53	3.8E-03	0.80	0.51	4.0E-02
high throughput	5.97	2.51	6.8E-04	0.39	0.21	6.6E-04
